# Author's Response to Peer Reviews of “Risk Factors of SARS-CoV-2 Infection: Global Epidemiological Study”

**DOI:** 10.2196/31910

**Published:** 2021-08-26

**Authors:** William Alphonse Barletta

**Affiliations:** 1 Department of Physics Massachusetts Institute of Technology Cambridge, MA United States

**Keywords:** COVID-19, pandemic, public health, mortality, infection, risk, risk factors, age, epidemiology, infectious disease

*This is the author’s response to peer-review reports submitted for the paper "Risk Factors of SARS-CoV-2 Infection: Global Epidemiological Study".*

## Round 1 Review

Author’s response to the review of the manuscript:

1. The Abstract and body of the text of the manuscript [1] have been restructured to conform with the standard format of the journal (ie, Background, Objective, Method, Results, Conclusions).
2. The revised text is based on the updated manuscript as recommended by the editor.
3. Figures that were suggested for deletion have been removed. All figures have been sized and reformatted per journal policy.
4. All footnotes have been removed, and the relevant information has been made part of the text or a numbered reference.
5. Equations are numbered for appropriate reference in the text.
6. I have added some quantitative values in the Results section of the Abstract.
7. I have removed the author-defined acronym, “CFR,” for apparent case fatality ratio.
8. Two references have been added in the Summary section.
9. The Summary and Conclusions have been expended to suggest potential effects of new SARS-CoV-2 variants.
